# Surgeon-Led Imaging Review for Patients with Periampullary Disease: An Important Aspect of the Preoperative Consultation

**DOI:** 10.1089/pancan.2018.0010

**Published:** 2018-09-01

**Authors:** Jessica A. Latona, Sami Tannouri, Theresa P. Yeo, Shawnna Cannaday, Harish Lavu, Jordan M. Winter, Charles J. Yeo

**Affiliations:** Department of Surgery, Sidney Kimmel Medical College at Thomas Jefferson University Hospital, Philadelphia, Pennsylvania.

**Keywords:** imaging review, pancreaticobiliary cancer, radiology review, surgical quality

## Abstract

**Background:** The perceived benefit of utilizing patients' own imaging studies as a preoperative educational tool has not been studied.

**Methods:** Pancreaticobiliary surgeons reviewed key findings of imaging studies with patients to educate about their diagnosis and inform treatment recommendations. Patient surveys were administered pre- and postvisit by an independent researcher to assess the impact of this practice.

**Results:** Only 55% of patients stated that it was important to see their imaging studies before the consultation. However, after the visit, 90% of patients understood their disease process better, and 86% of patients had a clearer understanding of their planned operation having seen their imaging studies. This represents significant improvement in patients' understanding of their medical condition (*p* < 0.05).

**Conclusion:** Reviewing imaging findings with patients is an underappreciated aspect of the surgical consultation. It is a powerful educational tool that takes little time, improves patient understanding, and enhances patient experience.

## Introduction

Imaging studies are a major component of the evaluation of the surgical patient, and of patients presenting with a pancreatic mass in particular. Obtaining these tests is costly and time consuming for patients. In addition, surgeons and their support teams may spend a great deal of time reviewing these images to determine if a patient is a surgical candidate and for surgical planning. Patients commonly read the reports or had key findings in the report communicated to them. In light of the importance of these studies to the overall management of patients, it is surprising that most patients make decisions without ever having seen their own imaging studies.

Numerous publications have addressed the appropriateness and timing of a radiologist reporting imaging results. The radiology literature has articulated the legal importance and moral incentive of communicating abnormal imaging findings in a timely fashion to patients.^1^ There is also a small body of literature describing patients' attitudes toward receiving imaging results. In general, when patients were polled, a majority expressed a desire for direct and fast communication of results even if it meant that the results would not be communicated by the ordering physician.^2–4^ While this discussion has centered on the communication of study results, very little is known about patients' attitudes toward a physician-led review of imaging studies with the patients and their family. There are several studies that have attempted to address the impact of reviewing images with patients or change patient behavior through the demonstration of personalized images, but the results have been inconsistent.^5–9^

Reviewing imaging studies directly with patients presents a multidimensional beneficial opportunity for surgeons to improve patient interactions and build a more trusting relationship. First, the process serves as an educational tool to help patients understand their medical condition and the rationale behind treatment recommendations. An informed patient is better equipped to make life-changing decisions. Second, it adds credibility to the recommendation, and builds trust between the patient and surgeon. Herein, we utilize direct patient surveys in an effort to gain a better understanding of the patient perspective and measure the impact of a surgeon-led imaging review at the time of initial surgical consultation. Our bias was that a surgeon-led review of imaging is appropriate, educational to patients and families, and has the potential to improve the utilization of a costly healthcare resource.

## Methods

Patients seen in consultation at the Jefferson Pancreas, Biliary and Related Cancer Center at Thomas Jefferson University Hospital from April 2016 through October 2016 were asked to participate in an institutional review board (IRB)-approved survey before and after being seen by a surgeon. All patients had previously had either a computed tomography (CT) or magnetic resonance imaging (MRI) of the abdomen and pelvis, and had been referred by their gastroenterologist or their primary doctor. The intent of the survey was to determine the impact of a physician-led review of the images during the consultation. The study used a random convenience sample based on the availability of personnel to administer the survey. The only inclusion criterion was the presence of a pancreatic mass; otherwise, there were no discriminating factors affecting study enrollment.

Surveys were administered by surgical residents who were not involved in the care of the patients. Patients were informed that their answers to the survey questions would not affect medical decision making in their case and that their answers would be anonymous, and they were not provided information on the specific purpose of the study. All images were reviewed with patients by the consulting surgeon exclusively on a video monitor in the patient examination room. The basic concept of MRI or CT technology and the orientation of cross-sectional imaging were explained. Anatomical landmarks were highlighted, and key findings related to their specific pathology were identified. Patients were provided time to ask specific questions. The time spent on imaging reviewed was measured inconspicuously by a resident with a stopwatch during the patient encounter. The same resident administered the previsit and postvisit surveys.

The previsit survey included four questions as shown in Table 1, which are presented using a standard 1–5 Likert scale, where 1 represents strongly agree and 5 represents strongly disagree unless otherwise indicated. The time the surgeon spent physically reviewing the imaging studies with the patient during the consultation was recorded for each encounter included in the analysis. The postvisit survey was either seven or eight questions depending on whether the patient was ultimately deemed to have a resectable lesion (Table 1). The pre- and postvisit questions took less than 5 minutes each to administer. In some instances when a face-to-face discussion was not possible after the surgical consultation, the postvisit questions were answered in a follow-up telephone call with the researcher.

**Table 1.** **Previsit and Postvisit Survey Questions Administered to Patients**

Previsit questions	Postvisit questions
1. I understand my disease.	1. I understand my disease.
2. I understand my planned operation.	2. I understand my disease better having seen my CT or MRI scan.
3. I have seen my CT scan or MRI scan before (yes or no).	3. (If resectable) I understand my planned operation.
4. Seeing my CT scan or MRI scan with my own eyes is important to me.	4. (If resectable) I understand my planned operation better having seen my CT or MRI scan.
	5. (If unresectable) The surgeon's review of my CT or MRI scan was important in helping me understand why I am not a candidate for surgery.
	6. The surgeon's review of my CT scan or MRI scan was worthwhile.
	7. The surgeon's review of my CT scan or MRI scan was complicated.
	8. The surgeon's review of my CT scan or MRI scan took too long.
	9. What aspect of your entire office visit was the most important to you? (write-in)

A Likert scale (1 = strongly agree, 2 = agree, 3 = either agree or disagree, 4 = disagree, 5 = strongly disagree) was used for previsit questions 1, 2, and 4 and postvisit questions 1 through 8. Question 9 was open ended.

CT, computed tomography; MRI, magnetic resonance imaging.

The primary end-point of the study was to determine if patients believe they have a better understanding of their disease after a surgeon-driven review of their imaging study. A secondary goal was to determine if patients believe they have a better understanding of the planned operation. Analysis of pre–postvisit responses was performed using a Wilcoxon signed-rank test. Statistical significance was considered for *p*-values <0.05. The calculated sample size based on the primary hypothesis was 50 patients. All statistics were performed with IBM SPSS Software 22 (IBM Corp., Armonk, NY).

Thirteen encounters were excluded from the analysis if one or more of the following conditions applied: (1) imaging studies were not actually reviewed with patients during the visit (*n* = 5), (2) the duration of the imaging review with the patient was not measured (*n* = 4), or (3) the answers to both the previsit questions and the postvisit questions were not obtained (*n* = 6). Three surgeons participated in the study (H.L., J.W., and C.Y.).

## Results

### Previsit survey and imaging review

**FIG. 1.**
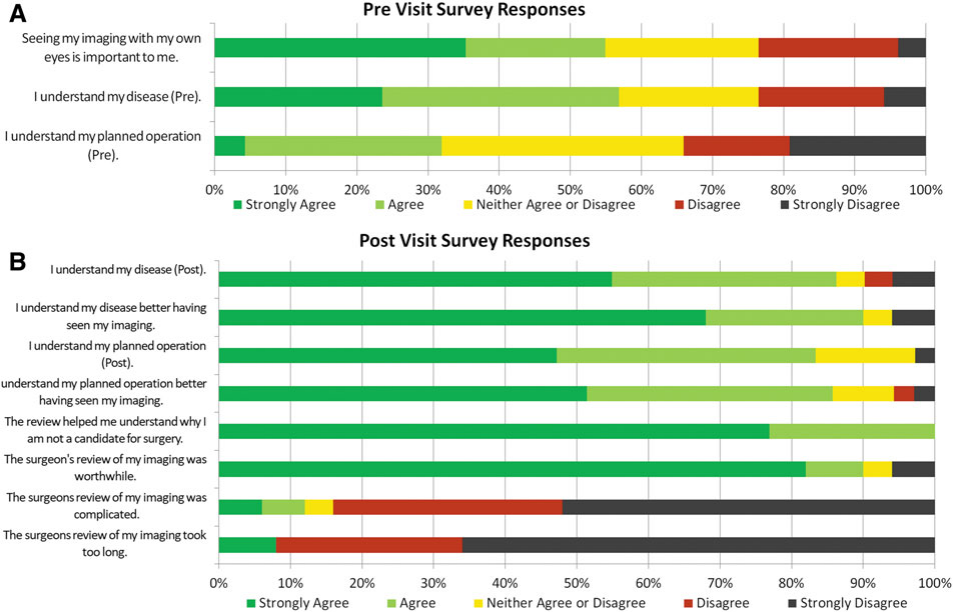
Patient responses to survey questions by Likert scale. Strongly agree to strongly disagree are depicted left to right. **(A)** This graph denotes answers given on the pre-visit questionnaire. **(B)** This graph denotes answers given on the postvisit questionnaire. Questions identical on both the pre- and postvisit questionnaires are labeled in the x-axis with a “pre” or a “post.”

The study included 63 patients, and 90% of previsit and postvisit surveys were completed. Thirty-nine patients (78%) had not seen their images before their surgical consultation. Eleven patients (36%) had reviewed their images on their own at home without physician guidance. Fifty-five percent of patients reported that seeing the actual images in addition to the radiology report was important to them before the consultation (Fig. 1A).

For the task of imaging review, the surgeons spent an average of 3 min 12 sec ± 1 min 42 sec (range: 34 sec to 8 min and 49 sec) individually going over the studies before meeting the patient. This does not include time spent by our experienced hepato-pancreatico-biliary nurse practitioners previewing images before the patient visit. The mean time for the surgeon-led imaging reviews was 2 min 42 sec ± 1 min and 51 sec (range: 31 sec to 11 min and 40 sec) explaining the imaging studies to the patients and their family members. The 2–3 min was the amount of time the surgeon spent physically scrolling through the imaging and explaining the anatomy and abnormal findings on the CT or MRI. There was time for discussion after and, if the surgeon paused for a question, this time was not measured.

### Postvisit survey

The patient responses to postvisit survey questions are displayed with the paired previsit question in Figure 1B. Patients reported that they understood both their medical conditions and the planned operations better after the consultation according to mean Likert scores. Specifically, the mean score for the statement “I understand my disease.” improved from 2.5 ± 1.2 to 1.7 ± 1.1 (*p* = 0.001). The score for the statement “I understand my planned operation.” improved from 3.2 ± 1.2 to 1.8 ± 0.9 (*p* < 0.001). Ninety percent of patients strongly agreed or agreed that they understood their disease better after having seen their imaging. Eighty-six percent of patients strongly agreed or agreed that they better understood the planned operation having seen their imaging.

Table 2 details a subgroup analysis based upon resectability of the pancreatic lesion. The patients were divided into four groups: those deemed resectable, those deemed unresectable and thus not surgical candidates, those with locally advanced disease or borderline resectable disease who were referred for neoadjuvant chemotherapy, and those who did not have a complete work-up at the time of the consultation. The table details the patients' response to the matching previsit and postvisit statement “I understand my disease,” the time spent reviewing the imaging with the patient, and the appropriate postvisit responses to the statements “I understand my disease better having seen my CT or MRI scan,” “I understand my planned operation,” “I understand my planned operation better having seen my CT or MRI scan,” and “The surgeon's review of my CT or MRI scan was important in helping me understand why I am not a candidate for surgery.” Notable findings include that for patients with locally advanced or borderline resectable disease, a much greater amount of time is spent reviewing the imaging. Specifically, for locally advanced or borderline resectable lesions, an average of 4.7 ± 3 min was spent reviewing imaging as compared with 2.5 ± 1.1 min for patients with resectable disease and 2.3 ± 1.8 min for patients who were deemed unresectable. This locally advanced or borderline resectable group also had a less favorable impact of the imaging review on the understanding of a potential operation as noted by minimal change in the average of the responses before and after the consultation. Due to the small size of the four subgroups, statistical analysis is not appropriate.

**Table 2.** **Subgroup Analysis of Patient Responses by Resectability**

	Resectable (*n* = 28)	Unresectable/nonsurgical candidate (*n* = 15)	Locally advanced/borderline resectability (*n* = 7)	Further work-up required (*n* = 3)
Previsit: I understand my disease.	2.4 ± 1.1 (2)	2.7 ± 1.3 (2)	2.9 ± 1.2 (3)	2.3 ± 1.2 (2)
Time spent reviewing imaging.	151 ± 65 sec (155 sec)	142 ± 106 sec (91 sec)	283 ± 177 sec (232 sec)	71 ± 15 sec (71 sec)
Postvisit: I understand my disease.	1.6 ± 0.9 (1)	1.9 ± 1.2 (2)	1.7 ± 1.4 (1)	2.3 ± 1.2 (2)
Postvisit: I understand my disease better having seen my CT or MRI scan.	1.5 ± 0.9 (1)	1.5 ± 1.1 (1)	2 ± 1.4 (2)	1.3 ± 0.5 (1)
If resectable: I understand my planned operation.	1.6 ± 0.7 (1)	N/A	2.8 ± 1.5 (2.5)	2 ± 0 (2)
If resectable: I understand my planned operation better having seen my CT or MRI scan.	1.6 ± 0.8 (1)	N/A	2.3 ± 1.6 (1.5)	2 ± 0 (2)
If unresectable: The surgeon's review of my CT or MRI scan was important in helping me understand why I am not a candidate for surgery.	N/A	1.25 ± 0.4 (1)	1 ± 0 (1)	1.5 ± 0.5 (1.5)

Data presented as mean ± standard deviation (median).

A Likert scale (1 = strongly agree, 2 = agree, 3 = either agree or disagree, 4 = disagree, 5 = strongly disagree) was used to quantify responses to the selected pre- and postvisit statements.

Overall, 90% of patients strongly agreed or agreed that the review of their imaging studies was worthwhile. A majority (88%) of the 23 patients who initially responded that seeing their images with their own eyes was not important strongly agreed or agreed that the review of their imaging studies was worthwhile. Only 8% of patients thought that the review took too long. Similarly, a small proportion (8%) of patients felt that the review of imaging was too complicated. Of note, all of the patients who were ultimately not deemed to be appropriate surgical candidates (*n* = 12) found the surgeon-led review to be worthwhile.

**FIG. 2.**
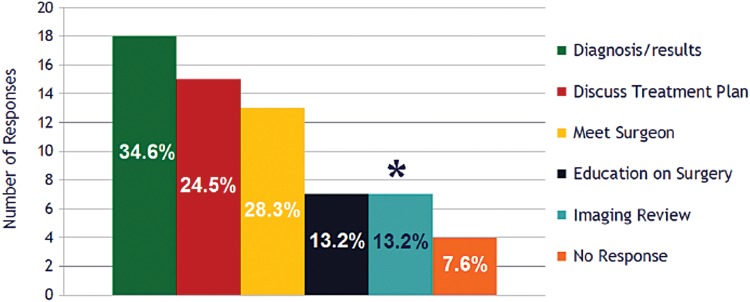
Patient responses to “What aspect of your entire office visit was the most important to you?” Asterisk highlights respondents who reported the imaging review was the most important aspect of their visit.

When asked an open-ended question to identify the most important aspects of their visit, 13% (*n* = 7) of patients indicated the surgeon-led imaging review. Responses to this question were grouped into the following categories and summarized as follows (Fig. 2): communication of study results or diagnosis (34% of respondents), discussion of the treatment plan (28%), meeting the surgeon (24%), discussion of surgical and postoperative expectations (13%), and the surgeon-led imaging review (13%).

There was no correlation between patient responses and the length of time spent reviewing images (*p* = 0.6); the patient being told he or she was a surgical candidate (*p* = 0.5); the diagnosis of malignancy, premalignant lesions, or benign lesions (*p* = 0.4); or the type of imaging (CT vs. MRI) that was reviewed (*p* = 0.06). Ultimately, 96% of patients who were appropriate surgical candidates and offered an operation underwent resection at the Thomas Jefferson University Hospital.

## Discussion

This is the first study to our knowledge that examines the impact of a physician-led review of patient imaging as an educational tool for patients and their families. Through a short survey of patient perspectives, our findings suggest the importance of this exercise in the evaluation of patients with a pancreatic mass.

First, patients' perspectives on the importance of viewing their imaging studies changed as a result of the review process. The overall percentage of patients who felt it was worthwhile increased from 55% to 90%. Indeed, 86% of the proportion of patients initially reporting that this was not important to them before the office visit ultimately changed their minds and felt that it was worthwhile. The impact was especially apparent for patients with unresectable disease. These data suggest that visual data may be useful in explaining to patients why they are not eligible at the time of consultation for surgery. In fact, published data across medical disciplines reveal improved comprehension,^10–17^ adherence,^18^ and satisfaction^17,18^ with the addition of visual information to patient–physician communications. This tool should be considered for what could be one of the most important conversations in a patient's life.

This may or may not hold true for patients who have locally advanced disease or are deemed borderline resectable. Interactions with these patients can be difficult, and require a nuanced discussion about the rationale behind neoadjuvant chemotherapy and possible post-treatment resection. The patients' responses are congruent with this. On the postvisit survey, this group had a split response to the statements that were designated “If resectable” and “If unresectable” (Table 2), indicating potential misunderstanding despite taking a longer amount of time explaining the imaging findings to patients. While they indicated that they understood their disease better than before the consultation, those who responded as though their disease was resectable had little change in their understanding of the planned operation. The imaging review did appear to have a positive impact on the understanding of the operation when patients understood that their disease was not immediately resectable and required treatment with neoadjuvant chemotherapy. One explanation for this finding could be that this subset of patients may have received a difference in opinion between the radiology report and the interpretation of the surgeon or other surgeons they had consulted with. These findings emphasize that this is a vulnerable group of patients who require added time for discussion during a preoperative consultation.

Second, the average time required for an effective surgeon-led imaging review was extremely short (less than 3 min), and virtually no patients felt this was too long or complicated. Taken together, these findings suggest that this approach offers an efficient and welcome method of relaying information to patients and their families. These two findings demonstrate an opportunity to align patients' demand of high-quality care with the healthcare system's expectation of physicians to see more patients in less time without sacrificing the quality of care.

In the current healthcare climate, performance measures are used to assess healthcare against recognized standards. The National Quality Strategy (NQS) led by the Agency for Healthcare Research and Quality (AHRQ) focuses on six priorities: safety, person- and family-centered care, communication and care coordination, effective prevention and treatment of illness, best practices for healthy living, and affordable care.^19^ This study suggests that a physician-led imaging review during the surgical consultation supports the mission of affordable care, in that it adds no additional cost. We would encourage surgeons to incorporate a short imaging review into their practice as it allows for value-focused care with an improvement in the utilization of a high-cost resource (radiographic imaging).

Patient satisfaction is an increasingly important metric for patient care, and several studies reveal the importance of patient satisfaction on quality outcomes.^20^ Improved patient experience impacts self-management, quality of life,^21^ adherence^22–24^ and outcomes.^25–29^ A positive patient experience has been associated with a lower risk of medical malpractice,^30–32^ higher rates of employee satisfaction,^33^ and increased patient loyalty.^34^ The surgeon has only minutes to establish rapport with patients and their family, and to earn their trust such that they are willing to place their life in a surgeon's hands. The imaging review offers an opportunity to demonstrate expertise, explain anatomy, illustrate pathology, and show that the surgeon is willing to spend extra time to enhance the patient's understanding. Communicating visually through these radiographic studies can help bridge a knowledge gap across patients, their families, and their physician. Such a practice emphasizes establishing an understandable care plan, encourages shared decision making, and therefore may enable patients, their families, and caregivers to manage their care more effectively. Though patient satisfaction was not specifically assessed in this study, our data indicate that the overall patient experience is enhanced by a surgeon-led imaging review.

The hypothesis driving this study was that reviewing images with patients and their families is an important and underappreciated aspect of the surgical consultation. We recognize that quantifying the impact is fraught with challenges. Moreover, there are several limitations related to the study design and execution, which could confound results. First and most importantly, it is our practice to review images with all patients making it unfeasible to have a control group of patients in this study. Perhaps, a future analysis will include a control group of image-naïve patients if such a practice is deemed ethical. Second, patients had varied degrees of understanding on their illness before the actual visit with the surgeon, and some had even received recommendations from other surgeons. Third, these results may be biased by variation in the timing of the previsit survey. Some patients were interviewed first by the researchers as compared with others who were interviewed after having met with other members of the care team who may have provided some education. Fourth, our inherent bias in favor of sharing their imaging studies with our patients may have influenced our interpretation of the data. Such limitations need to be recognized and may serve as a stimulus for further study.

In addition, it is entirely probable that aspects beyond the surgeon-led imaging review influenced postvisit survey responses. The postsurvey questions were answered by patients after a comprehensive consultation including meeting with multiple providers (often surgeons, nurse practitioners, and research coordinators) who delivered information as well as written resources regarding the diagnosis and care plan. Furthermore, the free text survey questions revealed that the majority of patients identified other aspects of the meeting that were most important for them, such as simply meeting their surgeon or receiving a diagnosis. However, when given the chance, multiple patients offered that the imaging review was actually the most important aspect of the consultation. Moreover, we included some questions in the survey that were specific to the imaging review to ascertain the role of this exercise on overall patient perspective.

We acknowledge that the greatest limitation of these results is the absence of a control group. Ideally, patients would have been randomized to either a surgeon-led imaging review or the absence of one. However, the surgeons believed this to be such an integral part of the initial consultation that such a trial design would have lacked equipoise in their view. It would have been potentially unethical to consent patients to a study where they may not be able to receive a review of their imaging. Finally, this study was small, performed at a single site, and included a narrow set of diseases that has the potential to limit the generalizability of our results. Though not specifically studied, we believe that these findings are translatable to other surgical subspecialties.

## Conclusion

In general, patients are unaware of the value of reviewing their imaging studies with their surgeon. However, patients who received this demonstration almost universally appreciated its value. A surgeon-led review of patient imaging improved the patients' perceived knowledge about their diagnosis and understanding of their treatment recommendation. The process lasted only a few minutes but substantially transformed patient experience. Based on these preliminary results, additional studies to measure the importance of this approach on patient education, satisfaction, and outcomes in the same disease process, as well as others, should be considered.

## Abbreviations Used

AHRQ: Agency for Healthcare Research and Quality
CT: computed tomography
MRI: magnetic resonance imaging
NQS: National Quality Strategy
